# The Practitioners' Bookshelf

**Published:** 1893-02-11

**Authors:** 


### THE PRACTITIONERS' BOOKSHELF.

### EMBRYOLOGY OF MAN AND MAMMALS.*

It becomes daily more imperative that the embryology of man should have a recognised place in medical education. At present the medical student hears a little of it in anatomy lectures, still less in physiology, and rapidly forgets it in the dissecting-room and laboratory. But now that attempts to explain the processes of life—of normal life or of life in disease—are giving way before that frank acceptance of empiricism called vitalism, embryological knowledge acquires a new importance. So long as it was thought that the direct relation of the structure of the body to the more important functions was understood, knowledge of anatomy might be held an adequate equipment for the physiology of health and disease. But now all function is being referred to vital properties of the living cell, and a full knowledge of the history of living cells must be carried into clinical work. Things which are alive must be studied separately, regarded as of their own kind, obeying their own laws, and for us, at least at present, isolated from the laws of physics and chemistry.

A continuous chain of cell life binds every adult tissue with the initial egg-cell, and the history of the specialisation of the embryonic cells into the adult tissues becomes a necessary part of medical knowledge. Already embryological knowledge has supplied interpretations of pathological conditions and abnormal growths, but it has yet to be applied systematically to the study of normal function. Moreover, so great an advance in our knowledge has been made in recent years that, apart from the new edition of *Quain*, it may be said that embryology does not exist in medical textbooks.

This book provides exactly what is wanted. For obvious reasons it has been impossible to work out the embryology of man as fully as the embryology of other mammals. Especially in the earlier stages, scarcity of material has left gaps. These Dr. Hertwig has bridged over by comparative embryology, and wherever the complicated processes of higher animals are by themselves unintelligible, Dr. Hertwig traces them back to simpler forms. The first part of the book deals with egg-cells and sperm-cells, the processes of fertilisation and cleavage, the formation of the germinal layers, the establishment of the external body form, and the nature and history of the fœtal membranes. Anyone who doubts the advantage to medical students of comparative embryology should convince himself by reading the sections on the latter subject. In the second part of the book the Hunterian method is followed, and the history of each tissue and organ is dealt with by itself.

The typography and illustrations are excellent, although some of the process reproductions have lost the charming softness that characterises the figures in the German edition. The translator deserves so well of English readers that it is enough to say that the German is accurately rendered.

* "Text-Book of the Embryology of Man and Mammals." By Dr. Oscar Hertwig. Translated from the Third German Edition by Edward Mark, Ph.D., Hersey Professor of Anatomy in Harvard University. (London: Swan Sonnenschein and Co., 1892.)

### MEDITERRANEAN WINTER RESORTS.*

This guide, which aims at being a practical handbook to the principal health and pleasure resorts on the shores of the Mediterranean, is the work of an experienced traveller. The information given in reference to nearly all the places described has been derived from a personal acquaintance with the various localities, but the whole volume being the work of one author, should be distinguished by greater uniformity of style. The editor has, however, increased the value of the book by securing the help of resident English physicians at the principal invalid stations, by whom the special articles on the medical aspects of these stations have been concisely, but authoritatively, stated. Though it is intended chiefly to meet the wants of that large class of persons who are advised to winter in the South, it will be found very valuable to medical practitioners who are called upon to select the particular region to which the patient should go in order to realise the best conditions for his peculiar case. We are glad to find that it is fairly well up to date, and that the spots which have only just recently grown into favour as health resorts have received special attention; but we think that it would be an advantage if, in future editions, this aspect of the work was further developed. The index is very incomplete, and we find that many of the less known stations are only to be found by looking under special headings, such as the "doctors" or the "churches." The map, which should be of great assistance, is of relatively little value here, being simply a portion of one of the publisher's Quarto Atlas series, in which the names of many places which are well known health resorts are difficult to discover amongst the crowds of places marked, while the majority of the less known seem to have been omitted altogether. A simpler map, specially designed for the book, would be preferable. But a book which has so rapidly run through its first edition has sufficiently proved its utility, and we do not hesitate to recommend all those who are intending to winter abroad to provide themselves with this handy little volume.

* "Mediterranean Winter Resorts." By E. A. Reynolds Ball, F.R.G.S. Illustrated by C. W. J. Prætorius. Second edition. (London: Edward Stanford. 1892.)

